# Chemical and Acoustical Mixed-Mapping of Geological Materials from Laser-Induced Plasmas: A Comprehensive Approach to Differentiate Mineral Phases

**DOI:** 10.1021/acs.analchem.4c05214

**Published:** 2024-10-17

**Authors:** Markéta Bosáková, Javier Moros, Pablo Purohit, César Alvarez-Llamas, Karel Novotný, Javier Laserna

**Affiliations:** a UMALaserLab, Departamento de Química Analítica, Universidad de Málaga, Jiménez Fraud 4, Málaga 29010, España; b Department of Chemistry, Faculty of Science, Masaryk University, Kamenice 5, Brno 625 00, Czech Republic; c Departamento de Química Analítica, Universidad Complutense de Madrid, Plaza de las Ciencias, Ciudad Universitaria, Madrid 28040, España; d Institut Lumière Matière (iLM), UCBL-CNRS, 10 Ada Byron, Villeurbanne 69622, France

## Abstract

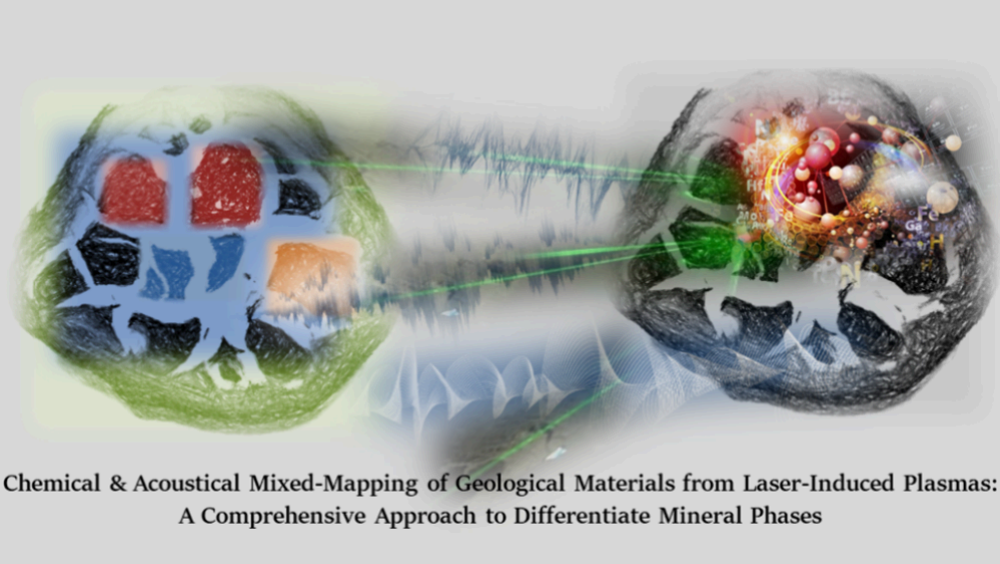

The acoustic wave produced alongside laser-induced plasmas can be used in conjunction with the recorded atomic spectra of plasma emission to expand the physicochemical information acquired from a single inspection event. Among the most interesting uses of acoustic information is the differentiation of mineral phases with similar optical responses coexisting in geological targets. In addition, laser-induced plasma acoustics (LIPAc) can provide data related to the inspected material's hardness, density, and compactness. In this paper, we present a dual acoustic–optic laser-based strategy for the generation of high-resolution surface images of mineral samples. By combining simultaneous multimodal LIBS (laser-induced breakdown spectroscopy) and LIPAc spectral data from laser-induced plasmas, we explore the mineralogical composition of rocks embedded in resin matrixes to distinguish their chemical composition as well as their crystal phases based on physical changes caused by the different spatial arrangements of the constituent atoms. The multispectral polyhedron created by merging singular optical maps, one per detected elements, and the coincidental acoustic map enhance the distinction between regions present within the matrix of a host rock as compared to the differentiation yielded by each technique when used separately. The chemical information guides the composition of the mineral phases in the host rock. Then, the physical information obtained from acoustics may reinforce the identification of the detected mineral phase, draw the geological history of the inspected section, and showcase possible transformations, mainly of polymorphic nature. To test the combination proposed herein, we also inspected a septarian nodule featuring an ensemble of mineral phases with different origins. Mixed optical and acoustic responses from laser-produced plasmas of this complex sample allowed us to obtain more specific information. This approach constitutes a reliable and high-throughput tool for studying the surface of geological samples, which can substantially supplement well-established techniques for mineralogical analysis such as Raman spectroscopy and X-ray diffraction.

Laser-induced breakdown spectroscopy (LIBS) has, in recent years, become a versatile tool for researchers striving to explore the chemical composition of an extremely diverse range of samples.^1^ In spite of the analytical performance shortages of LIBS, which are being solved by advance data acquisition and processing schemes, some of the exclusive advantages tied to the technique have motivated its gradual progress from conventional lab-based setups to its deployment within cutting-edge instrumentation systems.^2^ One of the most interesting traits of LIBS is its capability to work alongside other laser-based techniques, namely, Raman spectroscopy (RS),^3^ laser-induced fluorescence (LIF),^4^ or laser-ionization mass spectrometry (LIMS).^5^ LIBS shows great synergy with these techniques because they use similar equipment and do not require complex sample pretreatment. Hyphenated LIBS alternatives, operating either in single- or as multimodes, integrated in a switching device that allows the instrument to alternate between them, emerged to overcome major drawbacks found when employed individually. Many approaches have been proposed to make effective use of binary^3^ and even ternary^4^ combinations of these spectroscopic techniques in the field of material analysis for a variety of applications. Sets of spectral data sourcing from different high-throughput characterization techniques, capable of quickly yielding thousands of data points, may be correlated to provide more comprehensive descriptions of the samples under study within short amounts of time. In addition to the great number of data sets, the combinations considered above can often offer high spatial resolution. This fact has led to their application for the generation of bidimensional maps and hyperspectral images to study the microstructure and the distribution of chemical species on solids and their surfaces.^1,6,7^

LIBS has further advantages over the other laser-based techniques. A single inspection shot can provide direct and simultaneous dual information: the optical emission from the formed plasma and the acoustic emission from the snapping sound that accompanies the plasma.^8^ Many references in recent scientific literature lay a holistic view of the most interesting contexts for the use of acoustic waves derived from laser-produced plasmas.^9^ Some investigations have revealed that several parameters of the recorded acoustic signals are correlated to different physical traits of the ablated materials. Consequently, LIBS has the potential to combine light and sound for the generation of spectral images of materials, thereby prospectively starting a new active research area. Furthermore, if we think big, the attractiveness of LIBS and its associated acoustics for carrying out depth-resolved analyses of solids and probing the elemental distribution within may enable migration from physicochemical topography to its homologue tomography.

Although the topics for which LIBS has been used since its inception are very extensive, one field in which the use of the technique is experiencing a particular increase is geology, particularly in the mineralogy and petrology branches.^10−13^ A thorough characterization of minerals, sediments, and rocks, including traits such as size, location, morphology, and chemical composition, is of particular significance. Studying such factors is helpful to link the geomorphology and the biota controlling surface-level processes related to phenomena witnessed by the entity over the millennia.^14^ The magnitude of interest in this type of research is evidenced by its expansion beyond the boundaries of Earth. Laser-based instruments have already proven their outstanding capabilities for performing sophisticated characterization under circumstances where no other analytical technology can be deployed. The most contemporary example is SuperCam, a multispectroscopic instrument mounted on the Perseverance rover to explore the composition of geological samples on Mars as part of the NASA Mars 2020 mission.^15−18^ In the SuperCam instrument, for the first time, LIBS is associated with a microphone with the aim of acquiring off-lab data. Microphone data are expected to help discriminate mineral phases and palliate possible limitations that the rest of the techniques exhibit.^9^

The present work assesses the combination of optical and acoustic simultaneous spectral data from laser-generated microplasmas over the surface of geological specimen surfaces as a pathway to improve their characterization. By efficiently creating multielemental and acoustic images that map the distribution of the chemical species and the physical traits at the micrometer scale along large areas of the surfaces of geomaterials, enhancement of the discrimination of their mineral phases is pursued. Particularly, we strive to present a tool for categorization under extreme scenarios for which LIBS spectral responses differ only by minute variations in the relative intensity of the emission lines. Such is the case for polymorphs, mineral phases that contain the same chemical composition but exhibit differences in their physical properties.

## Experimental Section

### Experimental Setup

**Figure 1:**
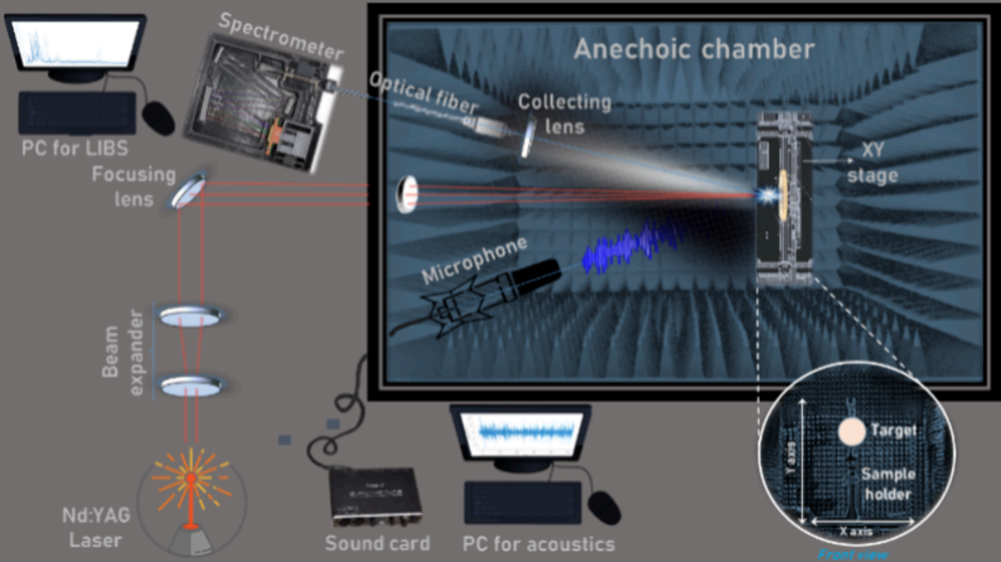
Plan view of the experimental setup of the tandem LIBS-LIPAc sensor. Inset (bottom right corner) shows in detail a front view of the sample arrangement for analysis. (Additional descriptive details are provided in the body of the text.)

We designed and assembled a contactless dual sensor that scanned across the surface of solid samples using nanosecond pulsed laser radiation to map their physicochemical profiles. A schematic draft of the system is shown in Figure 1. A pulsed laser (Ultra CFR Model, Big Sky Laser) generating 50 mJ pulses of 6.5 ns in length was used as the λ = 1064 nm light source for the ablation of the sample. Laser pulses were first expanded and then tightly focused onto the samples by a beam expander (consisting of a couple biconcave and plane-convex lenses with a focal distance of 25 and 100 mm) and a plane-convex quartz lens with a focal length of 600 mm, respectively. Using this configuration, 600 μm in diameter was decided as the maximum spot size on the surface of solid targets capable of resolvine optical and acoustical sample features at the laser pulse energy delivered. Within the LIBS-LIPAc framework of multimodal measurements from a single source, the excitation conditions need to be adjusted to a compromise situation that guarantees the best radiation intensity and shockwave snap sensitivity of the created plasma.

The samples were located 0.6 m away from the last focusing lens in a custom-built holder, which allowed for a single sample to be placed at once, fixed using double-side tape to keep its position, and mounted upon a set of two motorized linear stages to refresh the intrasurface sampling positions in the *X* and *Y* axes. The back of the sample holder was coated with neoprene foam for vibration dampening, thus mitigating the generation of interfering frequencies.

The emerging acoustic waves from plasma events were recorded at 96 kHz using a 6 mm prepolarized condenser microphone (20 Hz–19 kHz frequency response, omnidirectional polar pattern, 14 mV·Pa^–1^ sensitivity, TR-40 model from Audix). The microphone was placed quasi-coaxially—with an angle of about 10° to avoid blocking the light pathway—to both the laser light path and plasma expansion directions and at a fixed sample surface-to-microphone distance of 50 cm. A 24 bit/192 kHz audio interface (UA-55 Quad-capture model from Roland) was used at a sampling rate of 96 kHz for the digitalization of the acoustic waves. The Audacity software was used as the audio recording application.

The radiation from laser-induced plasmas was collected by a primary light-gathering convex lens used to focus the incoming light onto the tip of single 2m-optical fiber 600 μm core diameter. Plasma light was then guided to the entrance of a miniature Czerny–Turner spectrograph (AvaSpec-2048SPU2, from Avantes, equipped with a diffraction grating of 1200 lines/mm, 500 nm blazed, enabling a 250 nm spectral range, 0.4–0.6 nm spectral resolution -fwhm) with 75 mm focal length and fitted with a CCD detector. Using this configuration, LIBS spectra in the 310–550 nm window were obtained. Temporal settings to gather the plasma light were 1.1 ms gate width and 1.28 μs delay from the input of an external trigger supplied by the laser Q-switch output signal to activate the detector device.

The optical train for light collection, the microphone and the sample-holder were all housed inside a custom anechoic chamber (150 × 70 × 50 cm^3^, *L* × *W* × *H*) built with HiLo-N40 acoustic foam made of polyurethane with high rigidity and low density (70 mm total thickness, 40 mm knob height, 16.5 ± 1.0 kg·m^–3^ bulk density). The foam was used as an inside absorbent to reduce echoes and resonances as well external vibrations and noise. During operations, the room temperature was kept at 25 ± 2 °C.

### Samples

Investigations reported herein were conducted on naturally occurring materials found on Earth. First, natural forms of chalcopyrite (CuFeS_2_) (Rio Tinto mine, Huelva, Spain) and galena (PbS) (Osor mines, Girona, Spain) were chosen as defined crystalline structures representing the most important primary ores of copper and lead, respectively. Chalcopyrite is commonly found in a variety of geological environments, with widespread occurrence in nature in many countries around the world. Similarly, galena can be found in a variety of contexts such as hydrothermal veins (formed under various temperatures), metamorphic deposits, and pegmatites, with limestones and dolostones being common hosting rocks. Furthermore, it can be part, alongside chalcopyrite, of massive sulfide deposits associated with meta-volcanic rocks.

Then, two undifferentiated minerals—an aluminum-rich sedimentary rock (bauxite) (Zarzadilla de Totana, Murcia, Spain) and a sedimentary rock concretion (septarian nodule) (Jrada, Morocco)—were evaluated, both targets of no specific composition. Bauxites, the world’s main source of aluminum, are sedimentary rocks with a relatively high Al content. They consist mostly of hydrous aluminum oxides and aluminum hydroxides. Some of the minerals present in bauxites are gibbsite (Al(OH)_3_), boehmite (γ-AlO(OH)), and diaspore (α-AlO(OH)), mixed with some iron oxides goethite (FeO(OH)) magnetite (Fe^2+^Fe^3+^_2_O_4_), hematite (Fe_2_O_3_), and siderite (FeCO_3_), the aluminum clay mineral kaolinite (Al_2_Si_2_O_5_(OH)_4_), small amounts of anatase (TiO_2_) and ilmenite (FeTiO_3_ or FeO·TiO_2_), and other materials such as quartz. Septarian nodules are specimens where an outer shell of limestone—a relatively fine-grained soft sedimentary rock made up primarily of calcium carbonate, occurring as calcite and aragonite minerals—hosts concretions, commonly dominated also by calcite, aragonite, dolomite, barite, or siderite, with diverse structures and textures that evidence the multiphase character of the formation of these mineral aggregates.^19^ The septarian nodules are also interesting from a microbiological point of view. Concretions are confined bodies of clastic sediments lithified by authigenic minerals. The most common explanation of what induces the formation of the authigenic carbonate minerals in the concretions is microbial oxidation of buried organic matter under anoxic conditions. It is well-documented that cyanobacteria play important roles in the deposition of carbonates via biomineralization due to the production of extracellular polymeric substances.^20^ Thus, as microbes metabolize some component of the sediments, they change the local chemical balance, leading to the precipitation of the cementing minerals in the mud in quasi-spherical masses. The imbalance in the ratio of elements not only leads to significant chemical transformations but also the alteration of physical properties. This is why distinct dual spectral information from petrologically related concretions could be interpreted as a paleobiology proxy, distinguishing between an abiogenic and a biologic source related to the concretion formation.

Prior to their analysis, the specimens, originally in their natural form, were cut with a diamond saw given their irregular shapes (specimens ranged approximately between 4 and 7 cm across). Since the surfaces of the cut specimens' were not perfectly flat, they were smoothed using polishing paper. This smoothing alleviated surface roughness, which could alter the ablation process. The resulting fragments were embedded in epoxy resin using rigid cylindrical-shaped molds. Then, the excess resin was eliminated from one of the cylinder’s ends to expose the flat surface of the target. For analysis control, aluminum adhesive strips peripheral to the area to be interrogated were used as references.

### Data Acquisition and Data Processing

To precisely categorize geological material, the degree and types (compositional, distributional, and structural) of spatial heterogeneity need to be characterized. Thus, one of the main goals of the sampling approach is to reveal such heterogeneity at the greatest possible level of detail. This critical aspect boosts the significance of surface analysis techniques. A laser scanning was performed for high-resolution, two-dimensional point-by-point inspection of a well-defined reference grid on the geological specimen (subject to sample dimensions). Bidimensional scanning was conducted as multiple linear side-to-side tracking across the entire surface of the material, translating the target up the *Y* axis after completing each linear scan. At each linear side-to-side tracking (*X* axis), a strategy of three-quarters (75%) overlapping between successive pulses of spot size of ∼600 μm in diameter each was considered to enhance the image resolution. The scan lines were spaced 400 μm apart, resulting in a spatial resolution for the *Y* axis of 1 mm. The focal point was preserved to ensure identical ablation conditions during complete testing. Figure S1 in the Supporting Information presents an infographic on the interrogation of the surfaces, as well as a detail of the overlapping process of the successive pulses relative to each linear scan.

As a result, for each interrogated point on the sample surface, the optical emission and the acoustic spectrum of the associated snap were available for surface imaging. Then, the dual simultaneous spectral information reported by the plasmas generated over the grid was employed to build the 2D maps, providing the complete visualization of the surface physicochemical topography. Chemical maps, built by plotting the intensity of the characteristic wavelengths of atomic and ionic emission lines within LIBS data, provided in clear visual form information about the surface distribution of individual elements in the analyzed area. In parallel, an acoustic map was constructed using the absolute deviation for the most sensitive oscillation—named the peak-to-peak amplitude—of the sound wave. The reported value acted as a score for the plasmas’ sound assets associated with the spatial positions interrogated (see the information below in Figure S3 for more details). The resulting map informed on fluctuations in the physicochemical properties of the area. Thus, to emphasize differences between mineral phases, the strategy proposed herein is based on pixel-to-pixel image fusion by integrating multimodal orthogonal information from the same spatial coordinates. Orthogonal information exploits different principles, optical emission, and acoustic emission to deliver different parameters that elucidate the same analytical challenge. The goal is to compose and preserve in a single output image all the target information that is present in the multiple individual input images.^21^

The approach proceeds as follows: from produced plasmas, the spectral data allow us to extract multiple bidimensional outcomes of the scene. First, “monoelemental” maps from the optical emission intensity at any associated wavelength/s (either one or several) were generated. By using a moving-window algorithm, emission lines (complying to the 3σ criterion to be considered as detected) featured in the LIBS data set were assigned to their corresponding element by cross-checking with the information available in the NIST database.^22^ The number of chemical maps generated may be expanded to as many different elements as are identified. The more compositional heterogeneity the sample has, the higher is the number of maps. Second, we produced the “sound” map from the intensity of the time-domain acoustic emission main signal. Then, all maps were stacked to form a multispectral data polyhedron. Prior to integration, maps were individually normalized by scaling from 0 to 1. By doing this, biased contributions due to existing disparities between the emission intensity of the elemental lines (not necessarily linked to variations in the elemental content) and the inherent dynamic ranges of the LIBS and acoustic responses were minimized. Once the set of features that adequately described the content of the output image was determined, the observed intensity was deemed as the suitable metric for assessing the similarity between pixels. Hence, the higher the similarity between the scale values was, the more likely it was for the associated local regions to belong to the same entity.

## Results and Discussion

### An Overview of LIBS and Acoustic Data from Geomaterials

**Figure 2:**
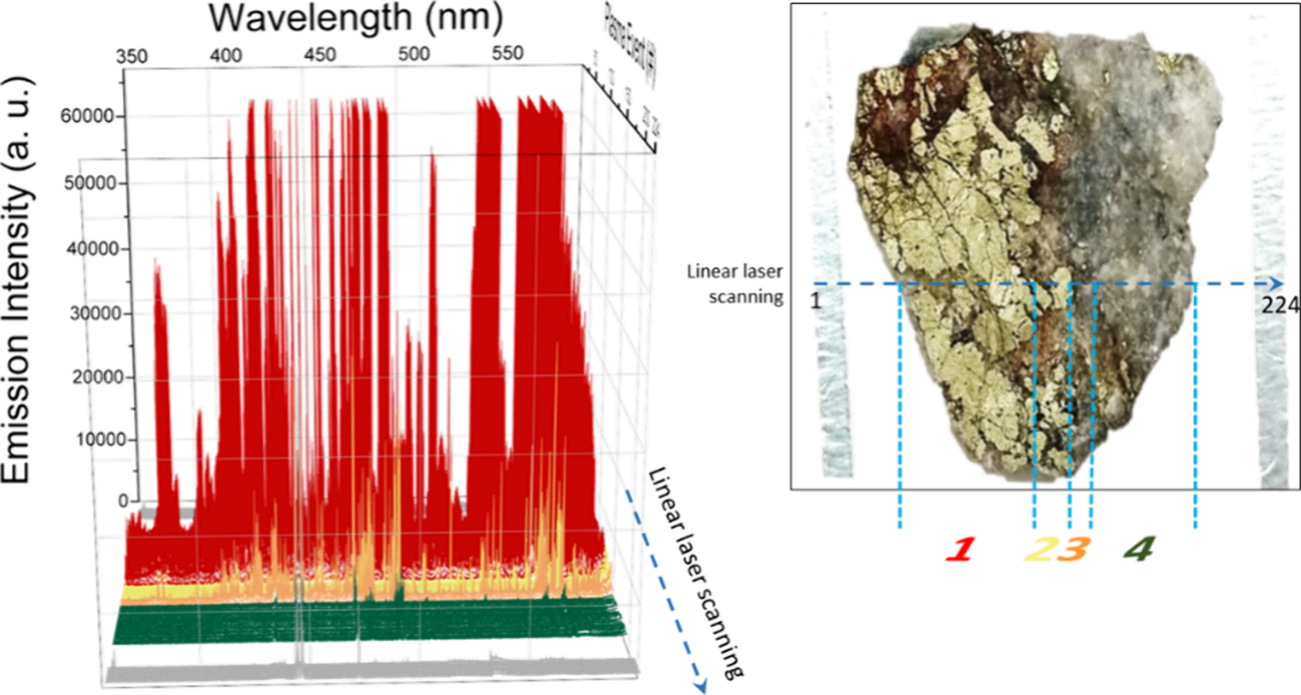
Left: LIBS response profile for a linear laser scanning (224 sampling points) over the surface of a chalcopyrite (CuFeS_2_) target. The blue dashed line guides the interrogated row. Right: photography of the chalcopyrite specimen evaluated. Vertical blue-dashed lines define the boundaries of the four well-defined batches of LIBS spectra.

Figure 2 shows the 224 LIBS spectra resulting from linear scanning over the surface of a chalcopyrite (CuFeS_2_) specimen.

The accompanying photograph of the sample shows that the chalcopyrite crystals were vastly surrounded by quartz crystals in the right section of the sample. The linear scanning profile allowed the identification of four well-defined batches of LIBS data, that is, sets of spectra of apparent high similarity in both type and intensity of their optical emission features. Batches are cataloged with a color code, namely, red, yellow, orange, and green. Although the spectral signals appeared different a priori for the first three batches, we found a matching degree larger than that perceived. Figure S2 in the Supporting Information compares the representative LIBS signals of each of those batches. An exhaustive cross-checking of the data evidenced that they featured emission lines belonging to the same chemical elements. The observed differences are justified by a changing detection sensitivity. The disparities found in the emission intensity are likely due to cracks intrinsic to the sample that remained after surface polishing. These alterations lead to a decrease of the ablation rate when compared to that of surface spots due to deviations from the focal point. Consequently, colder plasmas, with lower temperature and electron density, were produced at those spots, and the less sensitive spectral features were lost for these lower-intensity plasmas. Nevertheless, in agreement with the elemental composition of the mineral sample, LIBS spectra contained plenty of useful emission signals associated with a line-rich element like Fe, with those at 358.12 and 373.49 nm being the most relevant. Furthermore, lines related to Cu were also identified at 324.74, 327.40, 453.97, 458.68, and 465.01 nm. In contrast, in our data sets, sulfur emission signals could not be observed. This fact could be attributed to the poor intensity of the sulfur lines in the studied spectral range, which may also be masked by the many Fe-related lines. It is worth mentioning that this does not discard the possibility of S emissions being present outside the monitored wavelength window.

The large measurement-to-measurement uncertainty caused by the variability in texture, grain size, and surface roughness in naturally occurring mineral specimens can severely hinder quantitative elemental analysis and their classification. Our LIBS data alone suggested that the ore was Fe and Cu based. Nevertheless, it is complex to accurately determine the stoichiometric ratios that define the chemical formula and correctly categorize the mineral phase under analysis. In this context, several different chemometrics-based prediction approaches (which required a wide geologically relevant calibration suite) have demonstrated success for the quantification of elements.^12,13^

**Figure 3:**
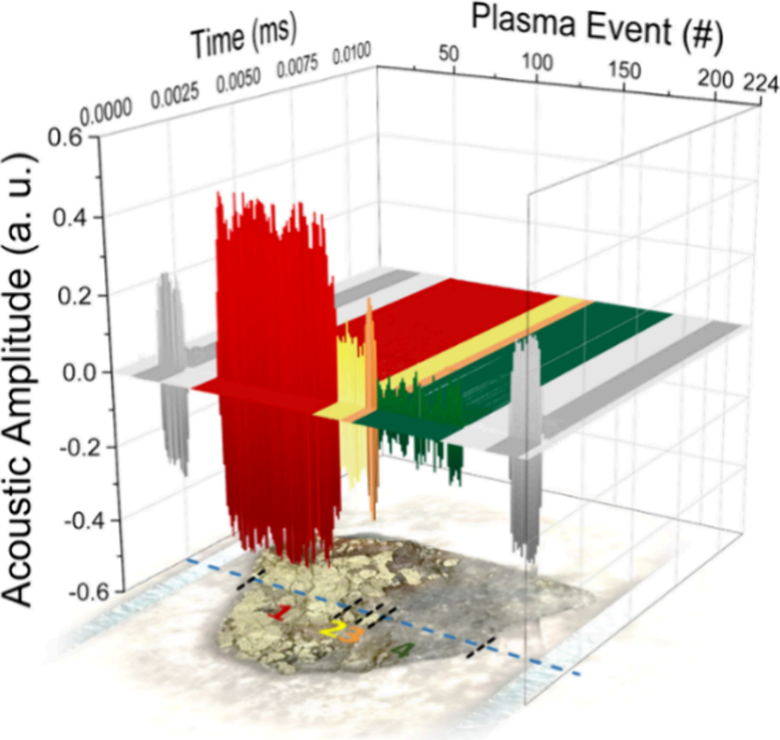
Single-row LIPAc response profile within the laser scanning raster (29 × 224 sampling points) over the surface of a chalcopyrite (CuFeS_2_) target. The blue dashed line guides the interrogated row. Black dashed lines define the boundaries of the four well-defined batches of time-domain LIPAc spectra.

This scenario becomes more challenging when the main mineral phase is accompanied by impurities, e.g., from elements replacing Cu, Fe, and S to secondary minerals in the form of sulfides, carbonates, oxides, or silicates due to nonspecific paragenesis. Particularly, here, this situation is identified through the LIBS data of the fourth batch in which the LIBS information significantly differed from that of the previous three cases. These compositional changes affected the shot-to-shot emission reproducibility at the micrometric scale. Likewise, Figure 3 presents the acoustic (LIPAc) responses associated with the 224 plasma events yielding the LIBS signals plotted in Figure 2.

As with LIBS data, plotting waveforms showed that the acoustic pulses associated with the laser-induced sparks also highlighted differences within the evaluated linear section. Whereas the waveforms of the acoustic signals were quite similar in duration for all the plasma events, the acoustic amplitude for the main N-peak in the waveform differed from one batch to another (see Figure S3 in the Supporting Information). The sound amplitude of the N-peak in the acoustic waves was found to be aligned with the intensity of the LIBS signals. Although the magnitudes of both responses did not follow a complete plasma-to-plasma matching, in general, the higher the intensity of the optical emission is, the louder is the acoustic response. As known, acoustic information per se does not directly reveal the presence of specific chemical species in the sample.^9^ However, it may recognize physical traits of the surface that guide the generation of the laser-induced stress waves toward the plasma ignition. These traits may comprise characteristics like hardness, luster, color, density, and surface imperfections such as cracks or pores. Consequently, the recorded peak sound pressures of acoustic waves from individual plasma sources may significantly depart from one another, even at sampling positions for which LIBS indicates highly similar chemical composition.

In summary, while multielemental LIBS information dictates the chemical composition of each sampling point, an individual spectrum does not allow accurate assignment of an identity to the specimen. The same holds true for acoustic information from plasmas. While it evidences interpositional variations for physical features, an independent acoustic outcome by itself does not provide enough information to label a specimen. Still, the fidelity with which the acoustic waves reproduced the scanned physical profile and their inherent correlation to the chemical info make them a potential analytical tool that may contribute to report a more complete and accurate description of the specimen.

### LIBS and Acoustic Mapping

**Figure 4:**
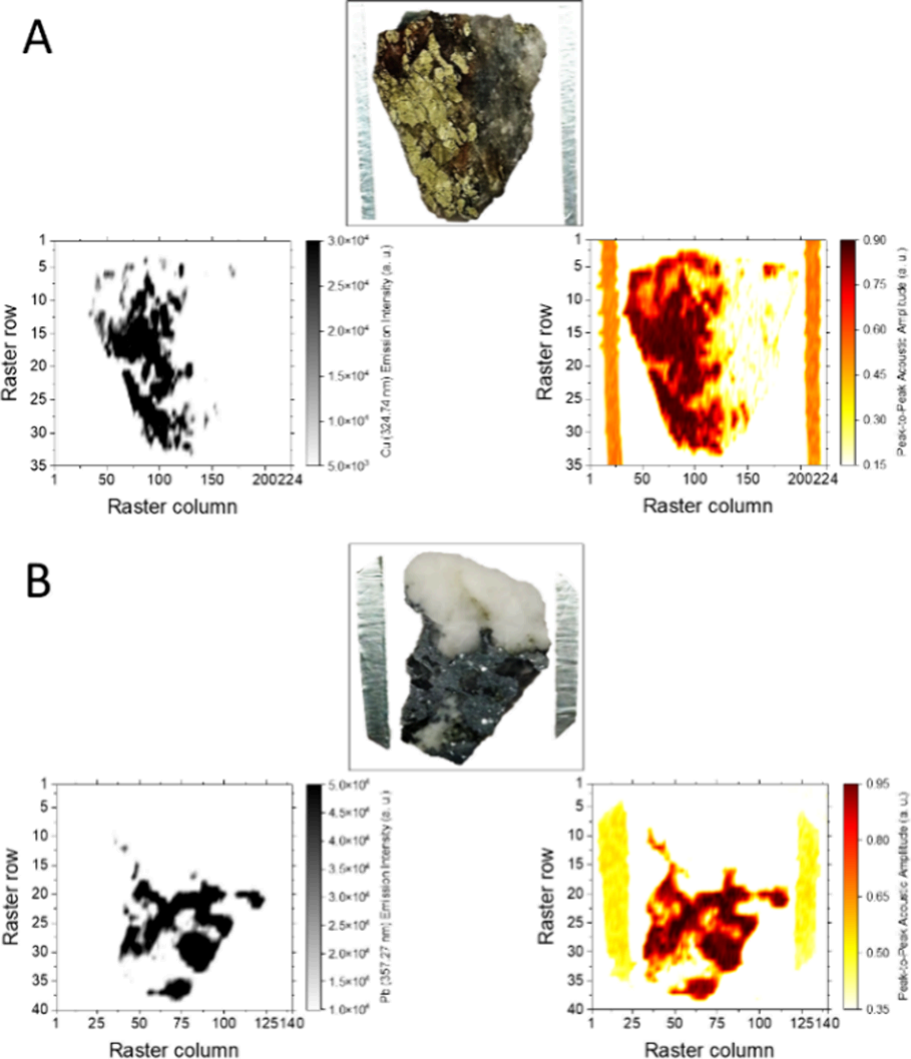
Elemental distribution map (left) and acoustic map (right) built from LIBS and LIPAc responses of plasmas induced over the surface of chalcopyrite (A) and galena (B) targets. Maps correspond to rasters involving 7840 (35 rows with 224 plasmas per row) and 5600 (40 rows with 140 plasmas per row) sampling sites, respectively. Chemical input data come from the intensity of atomic Cu, at 324.74 nm (chalcopyrite), and atomic Pb, at 357.27 nm (galena). Acoustic input scores resulted from the absolute intensity of the main N-peak in the sound waves.

Figure 4 shows the chemical and acoustic maps retrieved from the surface laser ablation of both the chalcopyrite (Figure 4A) and galena (Figure 4B) samples. Maps resulted from measurements on grids containing 7840 (35 rows with 224 pulses per row) and 5600 (40 rows with 140 pulses per row) sampling sites for chalcopyrite and galena, respectively. Chemical input data come from the intensity at the wavelengths of the most prominent lines of atomic Cu, at 324.74 nm, and atomic Pb, at 357.27 nm. Acoustic input scores resulted from the absolute intensity of the main N-peak in the sound waves. As shown, the maps, either chemical or acoustics, faithfully reproduced the surface profile of the specimen being evaluated. Chemical maps allowed visualization of the surface distribution of Cu or Pb along the surface. As a result, for both scenarios, a surface region missing the main constituent was identified, associated with secondary mineral phases, as discussed above. Similarly, the acoustic maps also provided evidence of compositional disparities. In addition, acoustic maps closely reproduced the physical alterations observed on the mineral surface, particularly cracks and voids.

These results suggested that combining both the optical and acoustic emission data could result in a positive synergy for cataloging mineral phases. Thus, sections sharing chemical content but sounding different (in the absence of obvious structural defects) or vice versa may hint at specimens that did not correspond to the same entity.

### Identifying Mineral Phases in Complex Matrices Using LIBS + Acoustic Information

The performance of the combination of optical and acoustic responses was evaluated for cataloging rock-forming minerals. Bauxite and the septarian nodule targets were chosen as complex samples for our proposed strategy. First, to validate the empirical picture of the bauxite herein interrogated, Figure S4 in the Supporting Information displays the X-ray diffraction (XRD) pattern on the mineralogical composition of some distinctive masses highlighted over the hosting background. The XRD analysis revealed that the investigated sample was mainly composed of kaolinite [Al_2_Si_2_O_5_(OH)_4_] (*d* = 2.65 g·cm^–3^), böhmite [γ-AlO(OH)] (*d* = 3.08 g·cm^–3^), diaspore [α-AlO(OH)] (*d* = 3.38 g·cm^–3^), grossular [Ca_3_Al_2_(SiO_4_)_3_] (*d* = 3.6 g·cm^–3^),^23^ and chlorite-serpentine minerals, whose general structural formulas may be expressed by A_4–6_Z_4_O_10_(OH)_8_ and A_2–3_Z_2_O_5_(OH)_4_, respectively, sheet silicates with hydroxyl anions at the exposed surface where “A” and “Z” in the formula represent cations of elements like Al, Fe, Mg, Mn, Ni, and Zn and Al, B, Fe, and Si, respectively.

**Figure 5:**
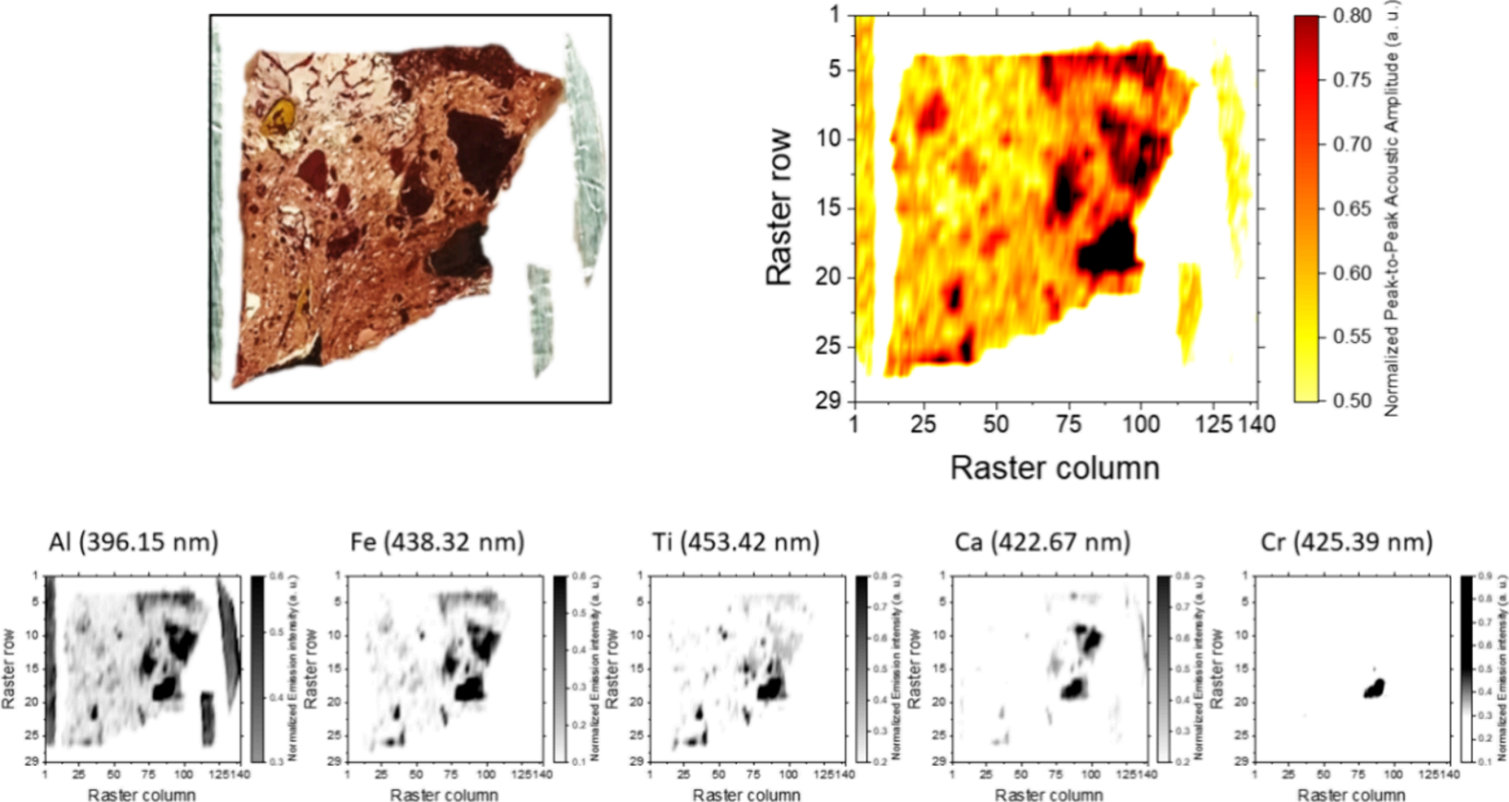
Acoustic map (top) and elemental distribution maps (down, at 1:2 scale) of Al (396.15 nm), Ca (422.67 nm), Fe (438.32 nm), Ti (453.42 nm), and Cr (425.39 nm), normalized all, built from LIPAc and LIBS responses of plasmas induced over the surface of a bauxite target. Maps correspond to a raster involving 4060 sampling sites (29 rows with 140 plasmas per row).

The composition and physical properties of these materials vary as these cations substitute each other in the crystal structure. Thus, Figure 5 presents the normalized acoustic and chemical maps, detailing the distribution of Al (396.15 nm), Ca (422.67 nm), Fe (438.32 nm), Ti (453.42 nm), and Cr (425.39 nm), built on the basis of 4060 sampling sites (29 rows with 140 pulses per row) on the surface of the bauxite fragment. As shown, the acoustic map highlights some irregularly shaped masses embedded in the host rock. Previous investigations on plasma acoustics from geological materials^24^ revealed that the lower the density of the mineral phase is, the lower is the acoustic energy of the shockwave recorded by the microphone. Based on the findings reported therein, we linked the masses present in the sample to mineral systems denser than the material forming the host rock. Differences were suggested as well by coincidental elemental chemical maps, yet the intensity of the optical emission itself did not suffice to discern between the host and occlusion. It is worth underlining that the strength of emission signals, regardless of the emitter, is conditioned by the matrix and its inherent ablation rate rather than by the amount in which an element is found. Thus, a low amount of element located in a region with a high laser ablation efficiency rate may lead to signals as intense as those coming from a low ablation rate over a high content of the same element. In other words, the conjunction of acoustic and elemental data is expected to enhance the identification of similarities between surface sections while highlighting disparities. This combined approach exploits the strengths of both data types.

**Figure 6:**
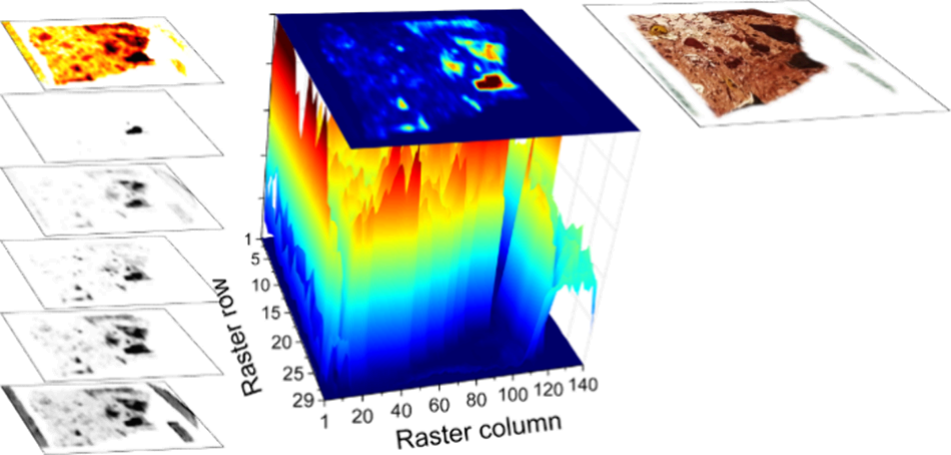
Spectral data polyhedron resulting from the stacking of the gathered maps, those associated with the “monoelemental” chemical distributions and the acoustic one, displayed as tomographic slices on the left side.

Figure 6 shows the multispectral data polyhedron resulting from the combination of the different “monoelemental” chemical maps and the acoustic map of the sample. Results confirmed that the concatenation of multiple layers of information allowed similarities to be grouped together while magnifying the differences. Guided by the polyhedron, we could confirm the rock surface to be largely homogeneous. Such an observation could fit with the formation of bauxite through the weathering and leaching of kaolinite, where iron and titanium could be present as discrete mineral species and/or in its structure substituting for Al or Si, imparting the identified reddish color.^25,26^ This observation was corroborated by the elemental distribution as well as the low acoustic intensity, which agreed with its low density.

**Figure 7:**
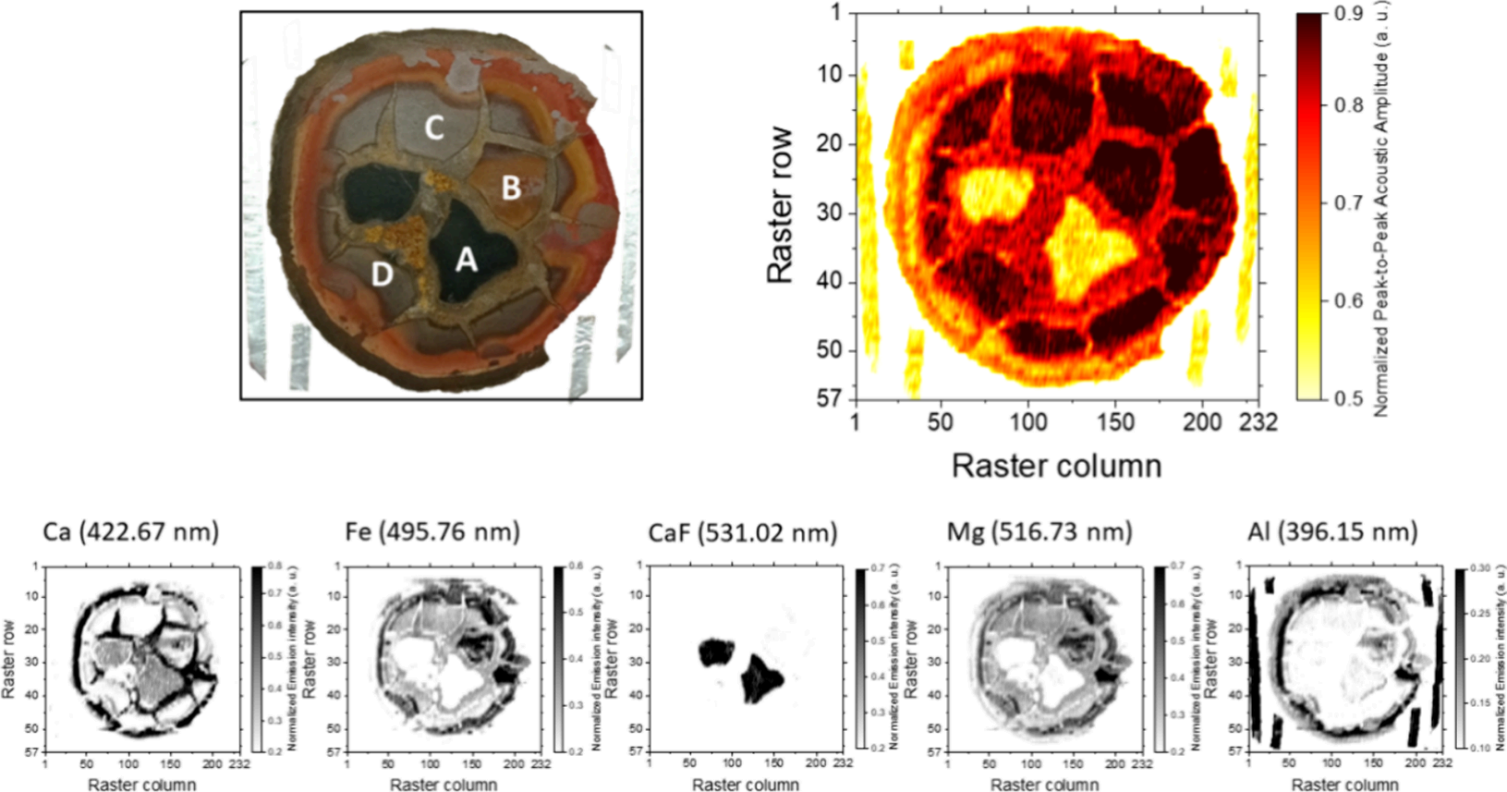
Acoustic map (top) and elemental distribution maps (down, at 1:2 scale) of Ca (422.67 nm), Fe (438.32 nm), F (monitored by the strongest band sequences of the B^2^Σ–X^2^Σ^+^ system for CaF bands located at 529–540 nm), Mg (516.73 nm), and Al (396.15 nm), normalized all, built from LIPAc and LIBS responses of plasmas induced over the surface of a septarian nodule. Maps correspond to a raster involving 13,224 sampling sites (57 rows with 232 plasmas per row). Distinctive polygonal blocks cataloged as A–D are labeled.

At first glance, some nodules stood out from the rest of the matrix, with one of them being more prominent than the rest. These nodules fit with the polymineralic nature of the rock, with iron oxides (böhmite and diaspore), grossular, and chlorite-serpentine minerals identified by XRD. Compared to kaolinite, the higher density reported for these phases complies to the direct relation found between density and sound intensity. Furthermore, their sourcing could be justified by the nonclastic nature of bauxite, which is formed through chemical routes rather than by mechanical processes. Thus, elements like Al, Ti, Fe, Ca, and Cr were enriched whereas others were depleted during the bauxitization process, with phyllosilicate minerals such as kaolinite being the main active phase.^27,28^ Crystallization of newly formed phyllosilicate minerals is strongly influenced by chemical microenvironments, with the cation ratio varying during the weathering and mainly depending on their crystal structure.^27,28^ In parallel, the stress induced by the self-organization of cations in the multiwalled structure generates changes in mineral density. The sum of these phenomena obscures the exact mineral composition of the masses involved. This is why, although these masses can be considered to originate from analogous systems, they are not strictly identical. The LIBS + acoustics combination strategy enhances the sensitivity to the disparities between these irregularly shaped masses embedded in the hosting rock. The suitability of the combined imaging from plasma light + plasma acoustics for the inspection of geological material was further tested by probing a septarian nodule with an area of approximately 40 × 43 mm, for which information from 13,224 plasma events was collected. Figure S5 and S6 in the Supporting Information display the Raman fingerprints and the XRD patterns, respectively, of the mineralogical composition for the most distinctive polygonal blocks of the nodule based on their chromaticism (cataloged as A, B, C, and D) separated by an inner rift constituting the bulk of the sample. As can be seen from Figure S4, the representative Raman spectrum of the rift between the blocks revealed a single, well-defined band at 1089 cm^–1^. This spectrum is consistent with the spectrum of calcite, CaCO_3_ (whose reported density is 2.7 g·cm^–3^),^23^ showing only the most sensitive feature related to the ν_*1*_(CO_3_)^2–^ stretching vibration.^29^ In contrast, representative Raman spectra from A blocks revealed a pair of well-defined features at ≈1600 and ≈1300 cm^–1^. These Raman shifts fit with the positions of the G-band and the D-band characterizing carbonaceous matter.^30^ The Raman band at 1594 cm^–1^ (O: order) is owed to the fundamental vibration of graphite, whereas the peak at 1305 cm^–1^ (D: disorder) is induced by structural disorder, and their intensities vary according to the level of structural distortion. Such information is corroborated by XRD data showing the corresponding relatively strong and broad diffraction peak (unresolved doublet) due to graphite (whose reported density is 2.26 g·cm^–3^)^23^ at about 26.75°. The XRD fingerprint of these particular blocks indicated also the presence of fluoroapatite [Ca_5_(PO_4_)_3_F], calcite, quartz [SiO_2_], goethite [FeO(OH)], and brindleyite [(Ni,Al)_3_(Si,Al)_2_O_5_(OH)_4_] in their compositional content. These results agreed with the data revealed by LIBS and acoustics featured in Figure 7, which show the normalized maps for Ca (422.67 nm), Fe (495.76 nm), F (monitored via the strongest band sequences of the B^2^Σ–X^2^Σ^+^ system for CaF bands located at 529–540 nm),^31^ Mg (516.73 nm), and Al (396.15 nm) and for the acoustic intensities of the associated plasmas. Apart from being coherent with the elemental content featured in LIBS spectra (with the exception of C and Si, which could not be detected within the spectral range under consideration), the lower density value associated with higher graphite content justifies the markedly less intense acoustics for plasmas recorded at A blocks as compared to acoustics associated with plasmas recorded at the rift.

**Figure 8:**
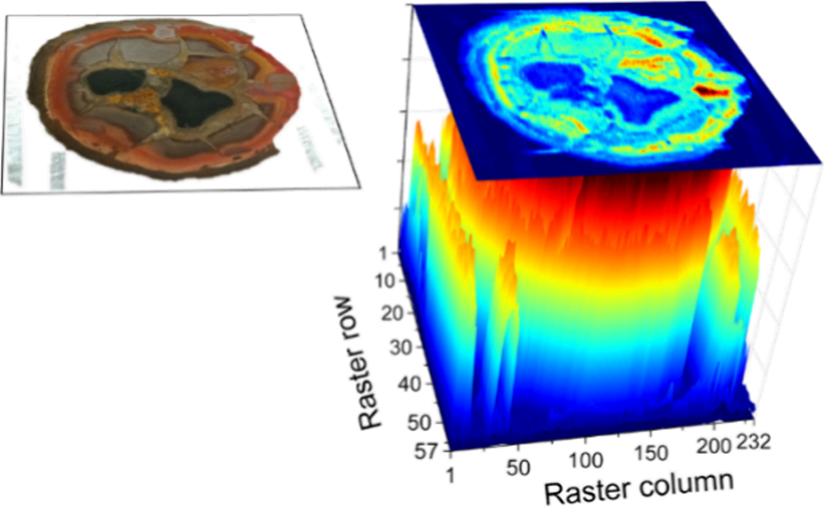
Spectral data polyhedron resulting from the stacking of the “monoelemental” chemical distribution maps and the acoustic map displayed in Figure 7.

In the case of the blocks B, C, and D, XRD and Raman analyses identified goethite (whose reported density is 3.8 g·cm^–3^)^23^ as the dominant mineral phase. Significant signals at 21.25, 33.3, and 36.7° within the XRD patterns were identified in Figure S6. Moreover, the intense band located at the ∼1800 cm^–1^ Raman shift (Figure S5) corresponded to the bending of the hydroxyl structure. In both signatures, it was possible to identify the presence of calcite (29.48° and 1089 cm^–1^, respectively) as well as a broadened band at ∼1300 cm^–1^ associated with residual defect density from the organic matter present in the sample. Results from both characterization techniques are a plausible explanation for the colors shown by the blocks, which varied from pale orange to light grayish. Naturally occurring chemisorption of metals to the goethite surface^32^ may be caused by episodic intrusions of aqueous solutions or due to successive sinking of the nodule into saline and alkaline groundwater, which may even spark new mineral phases. Meanwhile, carbonate cement progressively formed in the channels (rifts) created during the diagenesis of the sedimentary rock as CO_2_ escaped through them. This description matches the results revealed by the combination of LIBS and acoustic responses. The resulting multispectral data polyhedron is displayed in Figure 8. As seen, using this spectral combination, the A blocks stood out over the rest, being completely outlined due to their low optoacoustic response. It is worth mentioning that only in the multispectral polyhedron can physicochemical similarities between the A blocks and the light brown mineral region separating both block and the D block be found. Interestingly, despite looking identical to a grain located between the topmost part of the A blocks, this area showed a particular response. Focusing on this grain, Figure 7 shows that this feature yielded an acoustic response akin to that of the rest of the crack, whereas its emission was negligible from the optic point of view. This area was devoid even of the ubiquitously present Ca and Fe, with the former and Mg being the two elements responsible for its spectral activity and forming a ca. 0.4 intensity point in the polyhedron. Combining information from Figure 7, Figure S5, and Figure S6, we can confirm that this region, apparently part of the filling material at a first glance, turned out to be especially C-rich when LIBS + LIPAc was used. We hypothesize that the area possesses a singular geological history likely tied to the occlusion of microorganisms at one point in time.^33^ Moreover, the multisource stack also emphasized disparities between the B block and the C and D ones, which were more similar. In this case, the higher Ca content in the B block was responsible for the intensity increase in the middle-up region of the grain, which allowed the differentiation of a block otherwise similar to the bulk of the sample.

## Conclusions

We merged two of the leading-edge analytical techniques for off-lab geological analysis, LIBS and the recently proposed laser-induced plasma acoustics, to study compositional differences along the surface of rocks. From the plasma emission, we built maps showing how the different elements constituting the target were distributed. Then, using the peak-to-peak amplitude of the main peak featured in the acoustic responses when plotted in the time domain, physical traits of the inspected area such as density, optical absorption at the excitation wavelength, or compaction degree were evaluated. The acoustic information evidenced that sites leading to virtually identical LIBS spectra were, in fact, not identical. Thus, by combining data yielded from the optical and acoustic inspection of the samples, we were able to improve the differentiation of the different mineral species found embedded in the matrix of the hosting rock and provide a more accurate description of the mineral assemblages within the sample. We developed an acquisition scheme and a data processing routine that enabled the faithful reproduction of the surface of the material under study. This method facilitated tracing the particular history of the inspected grain, i.e., the physicochemical and biological phenomena experimented by a given occlusion in time, in contrast to the rest of the rock. Therefore, an accurate and fast surface tool for spectral imaging is introduced in the present paper, which we trust can transcend the mineralogical application reported herein and be used on a wide variety of multicomponent complex systems. Because, in the context of the planetary exploration programs and the search for life beyond Earth, science objectives encompass the reconstruction of the geological history of the surveyed landscapes and, through it, understand the past of the planet, extending this multispectral approach to evaluate its performance under extreme atmosphere conditions (composition, pressure, temperature) different from Earth’s as well as modeling acoustics in the frequency domain is now in progress.

## Supporting Information Available

Additional figures regarding comparative LIBS and acoustics spectra from laser-produced plasmas as well as XRD and Raman spectra of geological specimens (PDF)
