# Supplementary material for: Chemical and Acoustical Mixed-Mapping of Geological Materials from Laser-Induced Plasmas: A Comprehensive Approach to Differentiate Mineral Phases
Source: Anal Chem. 2024 Oct 17;96(43):17444–52. doi: 10.1021/acs.analchem.4c05214 (PMC11525928; doi:10.1021/acs.analchem.4c05214)

Chemical & Acoustical Mixed-Mapping of Geological Materials from Laser-Induced Plasmas: A Comprehensive Approach to Differentiate Mineral Phases

Markéta Bosáková[a,b], Javier Moros[a*], Pablo Purohit[c], César Alvarez-Llamas[d], Karel Novotný [b], and Javier Laserna[a*]

[a]*UMALaserLab, Departamento de Química Analítica, Universidad de Málaga, Jiménez Fraud 4, 29010 Málaga, España*

[b]*Department of Chemistry, Faculty of Science, Masaryk University, Kamenice 5, Brno 625 00, Czech Republic*

[c]*Departamento de Química Analítica, Universidad Complutense de Madrid, Plaza de las Ciencias, Ciudad Universitaria, 28040, Madrid, España*

[d]*Institut Lumière Matière (iLM), UMR5306, UCBL-CNRS, 10 Ada Byron, Villeurbanne, 69622, France*

***Correspondence:** laserna@uma.es

j.moros@uma.es

Table of Contents

Additional Figures........................... S2

Figure S1........................... S2

Figure S2........................... S3

Figure S3........................... S4

Figure S4........................... S5

Figure S5........................... S6

Figure S6........................... S7

Figure S1. Left: Infographic on the laser interrogation of the surface of a geological specimen through a multiple linear scanning. Right: Detail of the overlapping process of the successive laser pulses for each individual linear side-to-side tracking.

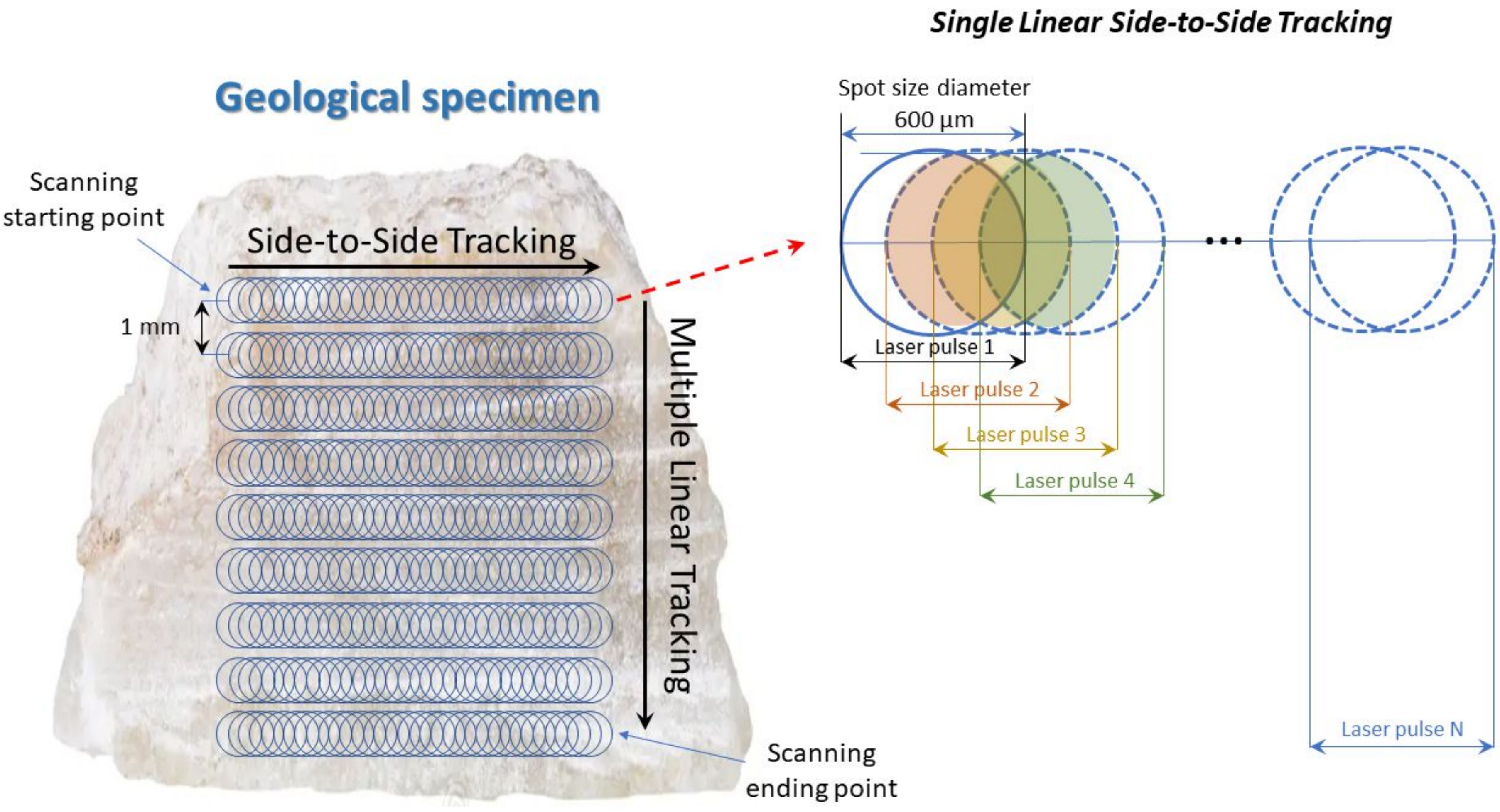

Figure S2. Comparison of the representative LIBS responses of the 3 first well-defined sets of spectra identified throughout the optical emission outcomes profile gathered for plasmas from the linear laser scanning over the surface of a chalcopyrite ($CuFeS_2$) target.

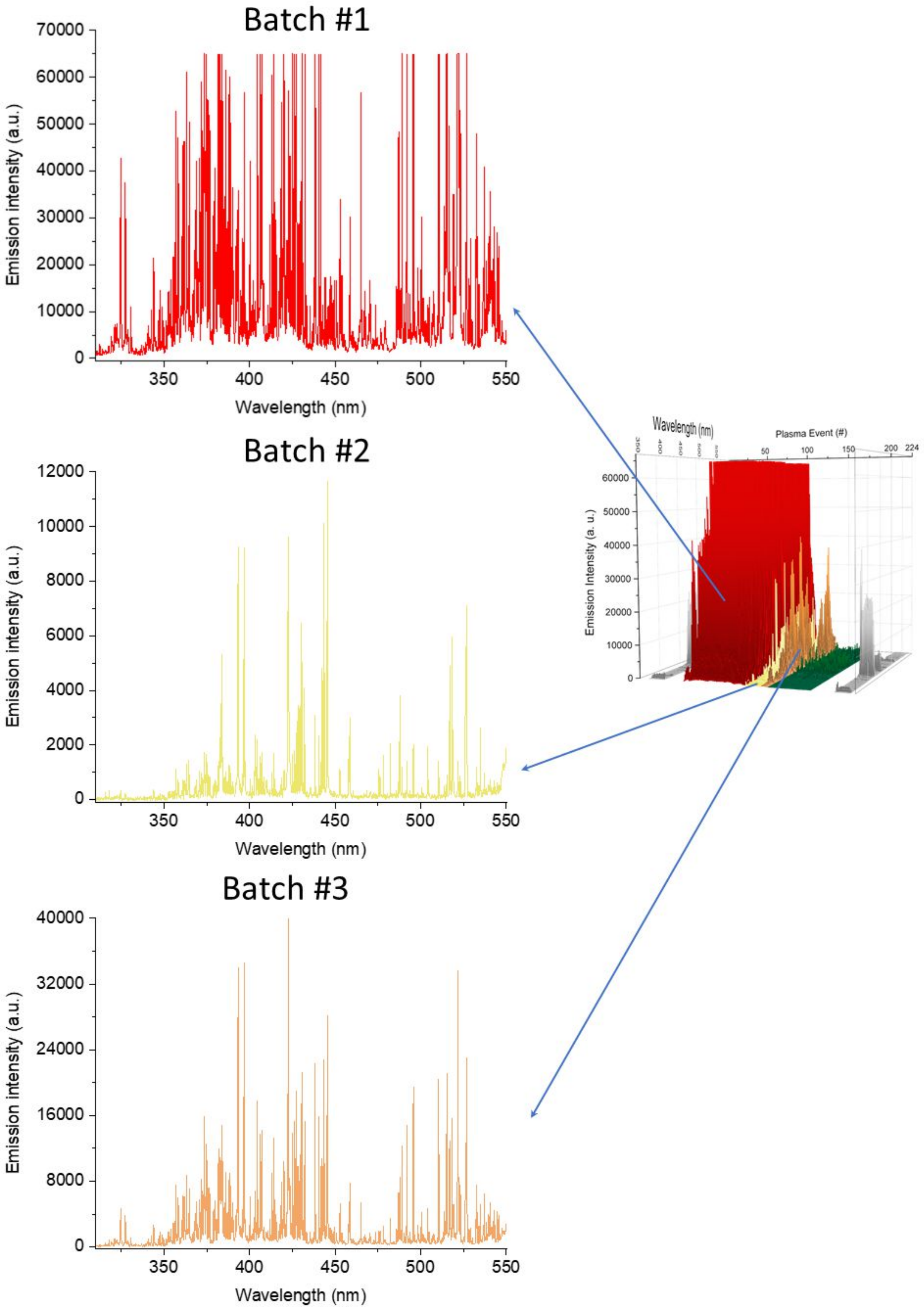

Figure S3. Comparison of the representative LIPAc signals of the 3 first well-defined sets of spectra identified throughout the optical emission outcomes profile gathered for plasmas from the linear laser scanning over the surface of a chalcopyrite ($CuFeS_2$) target. Blue-dashed lines define the boundaries of each set of acoustics spectra.

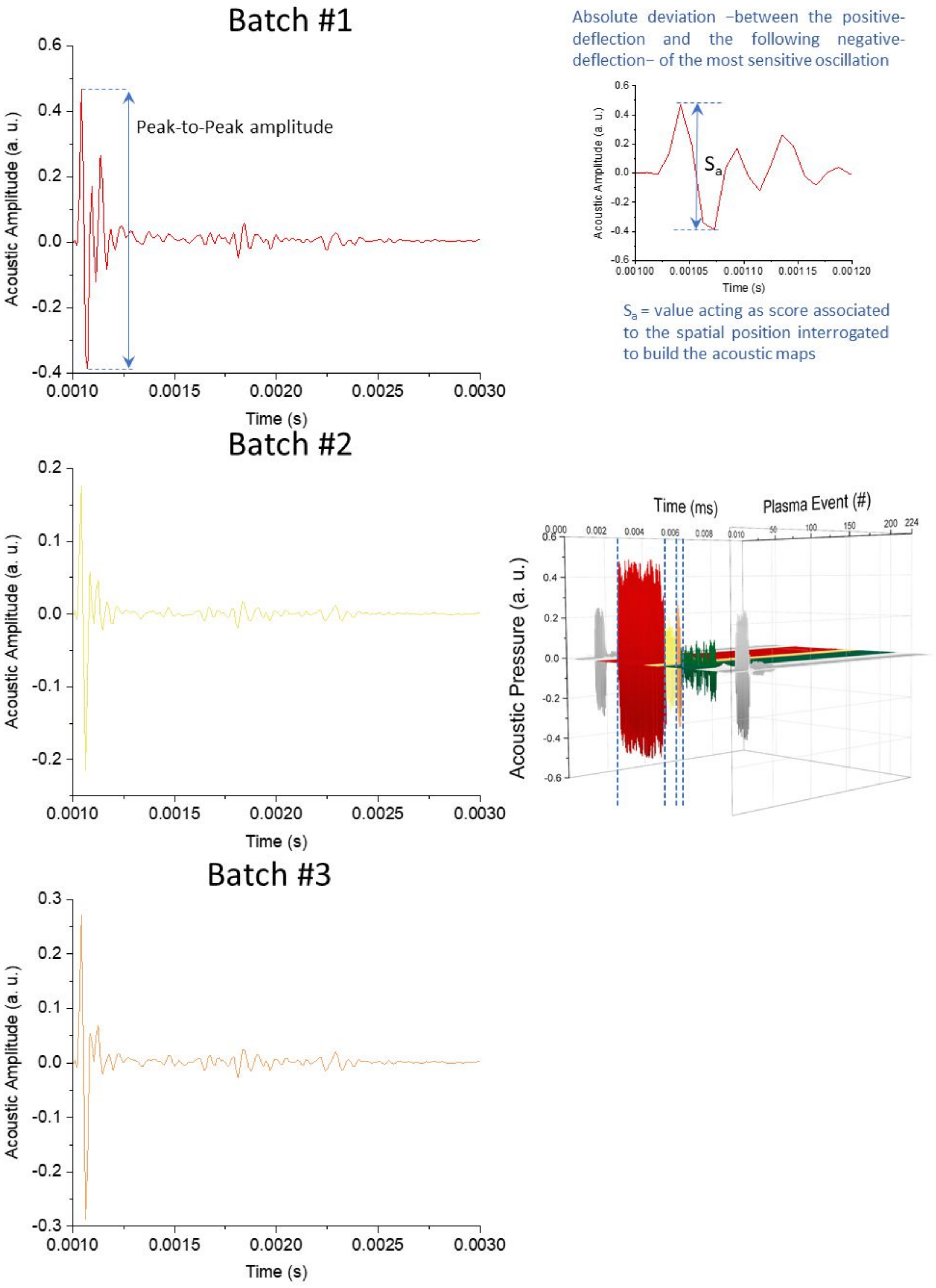

Figure S4. X-ray diffraction (XRD) pattern on the mineralogical composition of some distinctive masses highlighted over the hosting background surface of a bauxite target. XRD analysis was performed on a D5000 diffractometer (Siemens) Crystalline phases were identified by comparison of diffractograms with Powder Diffraction File database (PDF).

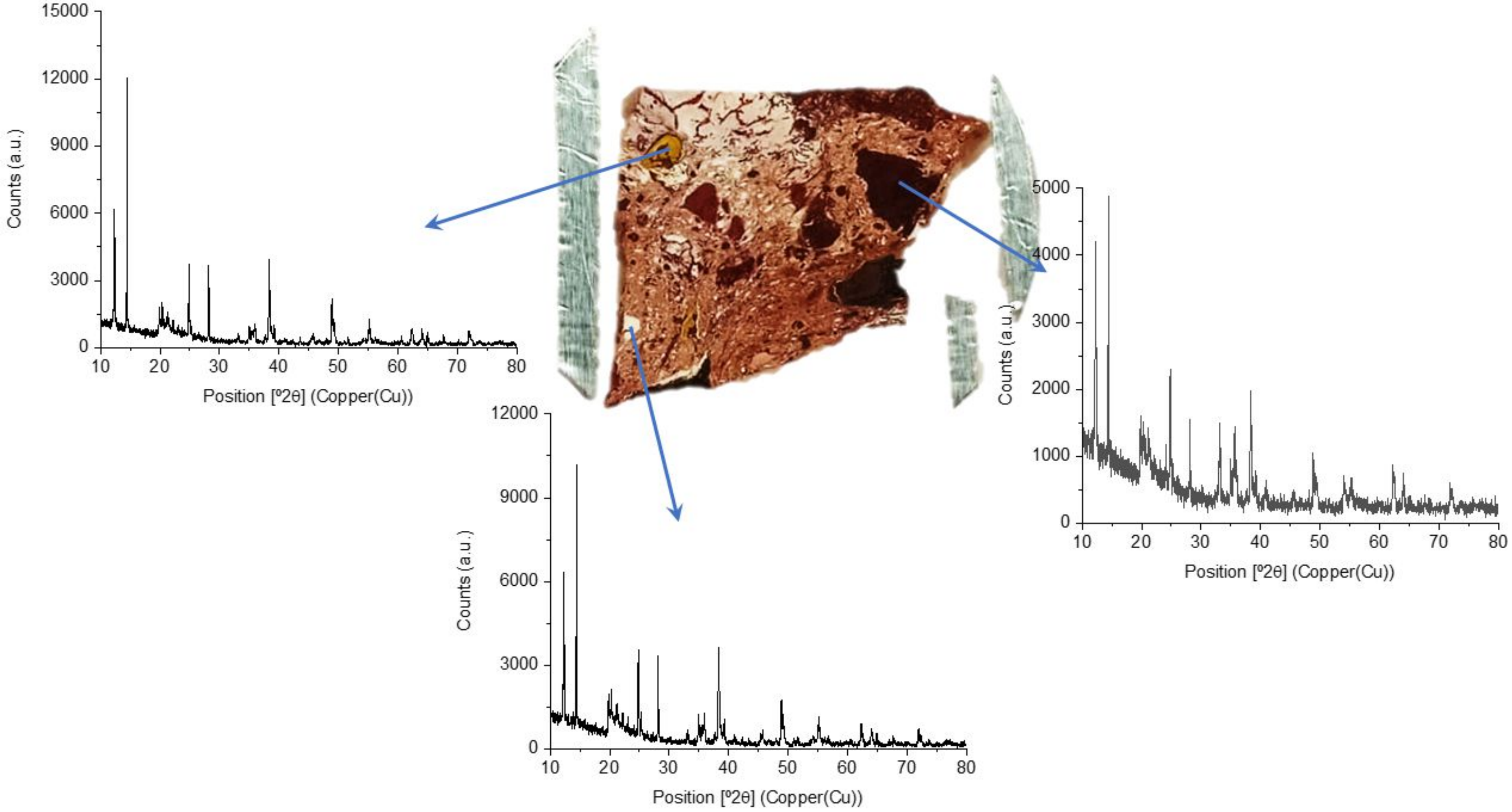

Figure S5. Raman fingerprints of some distinctive blocks highlighted over the hosting background surface of a septarian nodule target as well as of the crack filling intra-blocks. A DeltaNu Inspector 785 handheld spectrometer, which uses a 785 nm CW NIR laser with a maximum output power of 120 mW. This instrument covers a spectral range from 200 to 2000 cm^{-1} and has a spectral resolution of <8 cm^{-1}. The irradiance at the sample is ~0.7 $kW \cdot cm^{-2}$ in a 75 µm spot.

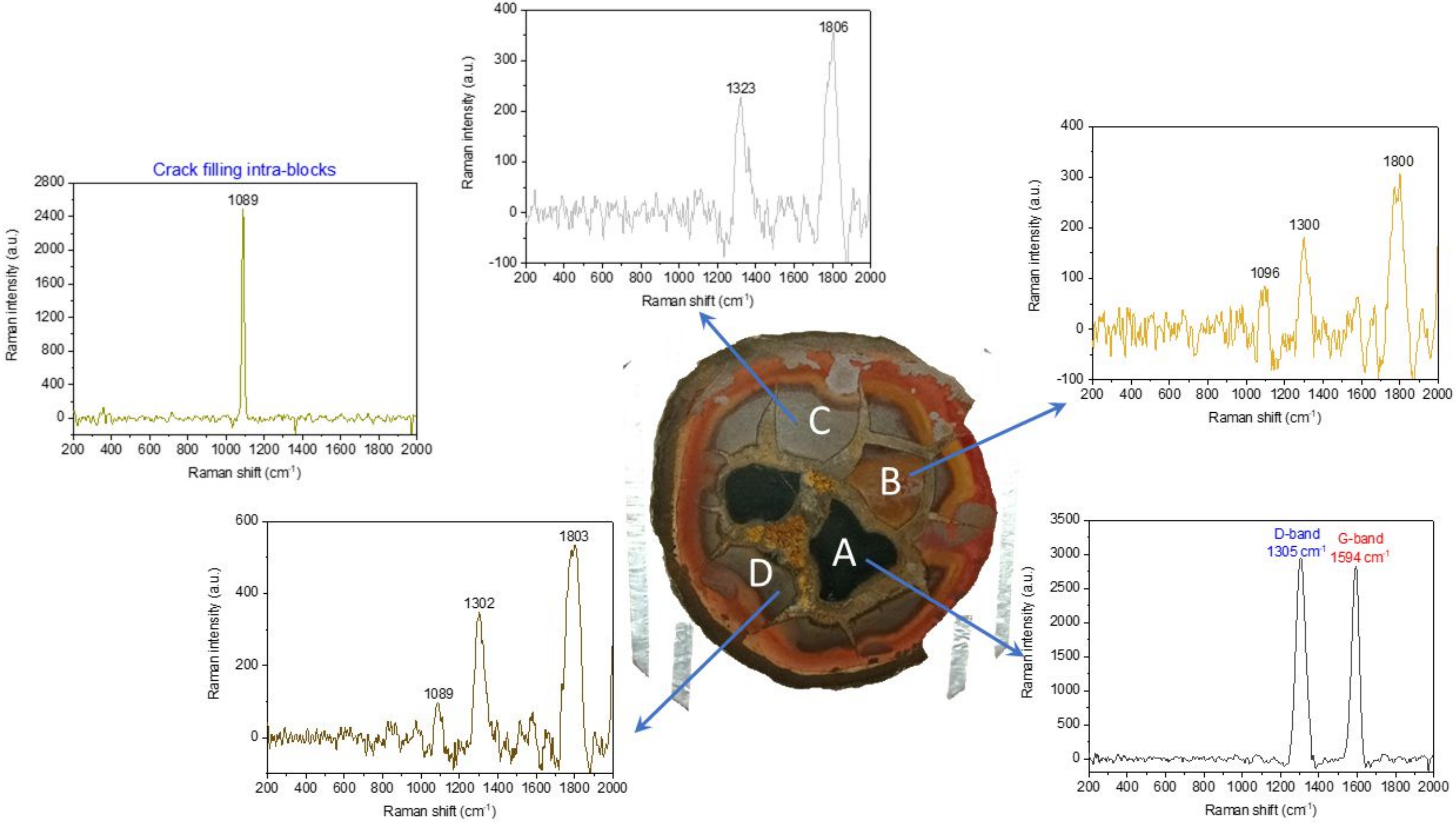

Figure S6. X-ray diffraction (XRD) pattern on the mineralogical composition of some distinctive blocks highlighted over the hosting background surface of a septarian nodule target. XRD analysis was performed on a D5000 diffractometer (Siemens) Crystalline phases were identified by comparison of diffractograms with Powder Diffraction File database (PDF).

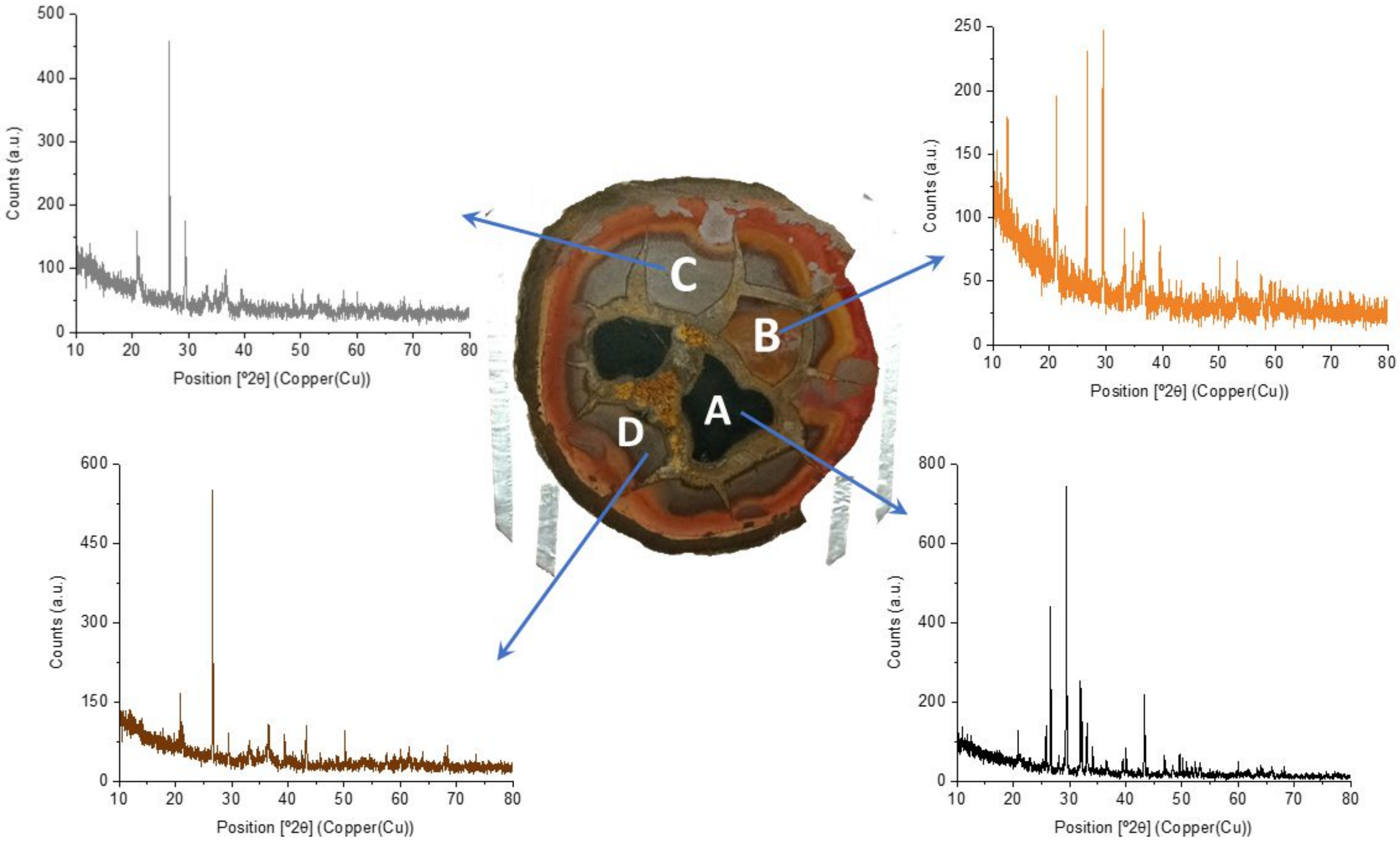

Supplement: Supplementary file 1 — ac4c05214_si_001.pdf [file ac4c05214_si_001.pdf]
